# Cord Blood-Derived Versus Homologous Donor Platelet-Rich Plasma (PRP) in Early Knee Osteoarthritis: A Single-Center, Randomized, Double-Blind Pilot Clinical Trial

**DOI:** 10.3390/bioengineering13070806

**Published:** 2026-07-14

**Authors:** Salvatore Alessio Angileri, Enrica Cristini, Simone Mazzola, Giovanni Maria Rodà, Chloe Yeabin Jung, Salvatore Valentino, Stefania Villa, Tiziana Montemurro, Larysa Mykhailova, Letizia Di Meglio, Simone Raoul Mortellaro, Vittoria Chiarpenello, Carolina Lanza, Roberta Gualtierotti, Daniele Prati, Gianpaolo Carrafiello, Luigi Piero Solimeno

**Affiliations:** 1 Department of Diagnostic and Interventional Radiology, Fondazione IRCCS Ca’ Granda Ospedale Maggiore Policlinico, Via Francesco Sforza 28, 20122 Milan, Italy; alessio.angileri@policlinico.mi.it (S.A.A.); letizia.dimeglio@policlinico.mi.it (L.D.M.); carolina.lanza@policlinico.mi.it (C.L.); gianpaolo.carrafiello@unimi.it (G.C.); 2 Division of Orthopedic Surgery and Traumatology, Fondazione IRCCS Ca’ Granda Ospedale Maggiore Policlinico, Via Francesco Sforza 35, 20122 Milan, Italy; enrica.cristini@policlinico.mi.it (E.C.); simone.mazzola@outlook.com (S.M.); turevalentino@hotmail.com (S.V.); luigi.solimeno@policlinico.mi.it (L.P.S.); 3 Postgraduate School of Diagnostic and Interventional Radiology, Università degli Studi di Milano, Via Festa del Perdono 7, 20122 Milan, Italy; yeabin.jung@unimi.it (C.Y.J.); simone.mortellaro@unimi.it (S.R.M.); vittoria.chiarpenello@unimi.it (V.C.); 4 Department of Transfusion Medicine and Hematology, Fondazione IRCCS Ca’ Granda Ospedale Maggiore Policlinico, Via Francesco Sforza 28, 20122 Milan, Italy; stefania.villa@policlinico.mi.it (S.V.); tiziana.montemurro@policlinico.mi.it (T.M.); larysa.mykhailova@policlinico.mi.it (L.M.); daniele.prati@policlinico.mi.it (D.P.); 5 Angelo Bianchi Bonomi Hemophilia and Thrombosis Center, Fondazione IRCCS Ca’ Granda Ospedale Maggiore Policlinico, Via Pace 9, 20122 Milan, Italy; roberta.gualtierotti@policlinico.mi.it; 6 Department of Pathophysiology and Transplantation, Università degli Studi di Milano, Via Festa del Perdono 7, 20122 Milan, Italy; 7 Department of Oncology and Hemato-Oncology, Università degli Studi di Milano, Via Festa del Perdono 7, 20122 Milan, Italy

**Keywords:** knee osteoarthritis, platelet-rich plasma, umbilical cord blood, regenerative medicine, intra-articular injection, cartilage regeneration

## Abstract

Intra-articular platelet-rich plasma (PRP) injections represent a promising biological treatment for early-stage knee osteoarthritis (KOA). As cord blood-derived PRP (CB-PRP) contains higher levels of bioactive mediators, this prospective study aimed to compare the efficacy and safety of CB-PRP versus homologous donor PRP (HD-PRP) in patients with early KOA and to evaluate whether CB-PRP results in greater clinical improvement. Fifty-one patients aged 46–70 years with Kellgren–Lawrence grade 1–2 were randomized to receive either CB-PRP or HD-PRP. Each underwent three intra-articular injections at four-week intervals. Outcomes included pain-related overall health perception assessed by the EuroQol Visual Analogue Scale (EQ-VAS), function measured with the Knee Injury and Osteoarthritis Outcome Score (KOOS), and quadriceps muscle strength evaluated by dynamometry. Assessments were performed at baseline and at 1, 2, 3, 6, and 12 months. Both groups showed improvements in pain and function over 12 months, with no statistically significant between-group differences (*p* = 0.46). EQ-VAS increased from 59 ± 19 to 83 ± 9 in the CB-PRP group and from 59 ± 18 to 77 ± 18 in the HD-PRP group. KOOS improved from 50 ± 25 to 68 ± 23 and from 38 ± 28 to 59 ± 24, respectively. Quadriceps strength showed no intergroup differences. Adverse events were mild and self-limiting. Both treatments demonstrated comparable clinical benefits and favorable safety profiles.

## 1. Introduction

Osteoarthritis (OA) is a degenerative joint disease characterized by progressive cartilage degradation and an irreversible clinical course that may ultimately require joint replacement surgery [1,2,3]. Although knee arthroplasty is effective for advanced knee osteoarthritis (KOA), conservative strategies are generally preferred in younger and middle-aged patients with early-stage disease to alleviate pain and maintain joint function [1,4,5].

In early KOA, articular cartilage may retain limited regenerative potential, emphasizing the importance of timely diagnosis and intervention [6,7]. Current non-surgical management includes pharmacological therapy, rehabilitation, and intra-articular injections aimed at symptom control and possible disease modification [1,8]. Corticosteroid and hyaluronic acid (HA) injections provide short-term symptom relief; however, their ability to influence disease progression remains uncertain [1,8,9,10].

Recent evidence has reframed KOA as a whole-joint disease involving not only cartilage degeneration but also synovial inflammation, subchondral bone remodeling, and disruption of the osteochondral unit [3,11,12,13]. This broader understanding has led to increasing interest in biological therapies as potential regenerative treatments for KOA, aimed at modulating the joint microenvironment and promoting tissue homeostasis rather than solely addressing symptoms [14,15]. Among these, platelet-rich plasma (PRP) has emerged as a promising option due to its potential to enhance tissue repair processes [16,17]. Recent systematic reviews and meta-analyses have suggested that PRP may provide superior and more sustained symptom relief compared with HA and other injectable therapies in selected patients with early-stage KOA [18,19,20].

PRP exerts its biological effects through a complex mixture of growth factors and cytokines involved in cell proliferation, chemotaxis, differentiation, and angiogenesis, including transforming growth factor-β (TGF-β), insulin-like growth factor-1 (IGF-1), vascular endothelial growth factor (VEGF), and platelet-derived growth factor (PDGF), as well as bone morphogenetic proteins such as BMP-2, BMP-4, and BMP-7 [21,22,23,24]. These mediators contribute to cartilage regeneration primarily by supporting mesenchymal stem cell recruitment and migration, as well as chondrogenic and extracellular matrix-forming activity [25,26,27,28].

PRP can be obtained from autologous or allogeneic sources, such as peripheral blood or umbilical cord blood. Autologous PRP, however, is subject to substantial inter-individual variability related to patient characteristics and preparation methods [29,30,31,32,33]. This has prompted interest in standardized allogeneic formulations derived from selected donors, which may improve consistency of biological activity.

Cord blood-derived PRP (CB-PRP) represents one such formulation and has been reported to exhibit a distinct cytokine profile, including higher levels of interleukin-10 (IL-10), which may contribute to modulation of inflammatory responses and eventual pain reduction [1,11,34,35]. Experimental studies have further suggested that CB-PRP may promote greater cellular responses than adult homologous donor (HD)-derived preparations [36,37,38].

Although both HD-PRP and CB-PRP are standardized allogeneic platelet products, they differ in biological origin and molecular composition. HD-PRP represents a more readily available source of platelets, which may facilitate broader clinical implementation, whereas CB-PRP is characterized by a richer profile of regenerative and immunomodulatory mediators but is potentially limited by donor availability. In vitro evidence indicates that CB-PRP contains significantly higher concentrations of growth factors than HD-PRP and has been associated with enhanced cell proliferation and migration [36,39]. Moreover, CB-PRP has demonstrated greater immunomodulatory activity by promoting the polarization of activated macrophages toward an anti-inflammatory phenotype and reducing interferon-gamma (IFN-γ) release [34,39]. Collectively, these findings suggest that CB-PRP may possess superior regenerative and immunomodulatory potential compared with HD-PRP, although clinical evidence directly comparing both formulations remains limited. Importantly, despite the increasing clinical use of allogeneic PRP products, no randomized controlled trials (RCTs) have directly compared HD-PRP and CB-PRP in KOA. Consequently, it remains unclear whether the biological peculiarities of CB-PRP translate into clinically meaningful advantages over conventional donor-derived PRP.

Given the higher concentrations of bioactive cytokines and growth factors reported in CB-PRP compared with adult donor-derived preparations, we hypothesized that CB-PRP may result in greater clinical improvement than HD-PRP in patients with early KOA. Specifically, in this single-center, randomized, double-blind pilot trial, the primary hypothesis was that CB-PRP would be associated with a greater reduction in patient-reported symptom burden at 12 months, as measured by the EQ-VAS, compared with HD-PRP. Secondary hypotheses included greater improvements in knee function (KOOS), quadriceps muscle strength, and overall symptom trajectory, while maintaining a comparable safety profile. Accordingly, this study aimed to compare the efficacy and safety of intra-articular injections of HD-PRP and CB-PRP in patients with early-stage KOA and to investigate whether potential biological differences between the two formulations are associated with differential clinical responses.

## 2. Methods

### 2.1. Study Design

This prospective study was approved by a local ethics committee (protocol n. 138406; ORTO PRP-KNEE 01/2021), conducted in accordance with the Declaration of Helsinki. This single-center, randomized, double-blind pilot clinical trial aimed to assess the efficacy and safety of umbilical CB-PRP versus HD-PRP in patients diagnosed with early KOA. Fifty-one patients with KOA were consecutively screened between August 2021 and March 2024. All patients provided written informed consent prior to enrollment.

Being exploratory in nature, this study aimed at evaluating the feasibility and preliminary effectiveness of the intervention. Consequently, no formal a priori sample size or statistical power calculation was performed, and the findings should be interpreted as hypothesis-generating, intended to generate preliminary data for future adequately powered confirmatory trials.

### 2.2. Patient Selection

Eligible participants were adults aged 46–70 years with symptomatic KOA classified as Kellgren–Lawrence grade 1 or 2, a body mass index (BMI) below 30 kg/m^2^, and insufficient clinical response to anti-inflammatory or analgesic therapy during the preceding three months. No participant received intra-articular HA injections before enrollment or during the study follow-up period.

Exclusion criteria included severe KOA (Kellgren–Lawrence grade 3 or 4); concomitant severe coxarthritis; inflammatory arthritis; clinically significant knee joint effusion; hematological disorder; ligamentous instability; mechanical malalignment exceeding 5° (varus or valgus); immunodeficiency; systemic corticosteroid therapy or local corticosteroid injection within the previous 3 months; positive infectious disease screening; active or prior oncological disease; and current anticoagulant therapy.

### 2.3. Randomization and Blinding

Data management and treatment allocation were performed using REDCap (Research Electronic Data Capture, version 11.4.4; Vanderbilt University, Nashville, TN, USA). Eligible patients were randomly assigned in a 1:1 ratio to receive either CB-PRP or HD-PRP according to a computer-generated block randomization scheme with a fixed block size of four; this was generated by an independent statistician not involved in patient recruitment or treatment administration, using R Statistical Software (version 4.3.1; R Foundation for Statistical Computing, Vienna, Austria). This approach ensured balanced allocation between the two treatment arms throughout the enrollment period while minimizing selection bias. Each participant then received a unique identification number linked to the predefined allocation schedule.

The study followed a double-blind design, with both patients and treating physicians blinded to treatment allocation. Allocation concealment was ensured by restricting access to the randomization sequence to authorized Blood Transfusion Center personnel responsible for product preparation, thawing, and dispensing, who were not involved in patient recruitment, treatment administration, clinical assessments, or outcome assessment. Before administration, both CB-PRP and HD-PRP were transferred into identical sterile syringes containing the same injection volume (10 mL) and labeled only with a study identification number, without indicating the source of the PRP preparation. Consequently, neither patients nor treating physicians were able to identify the assigned treatment. The allocation code was securely maintained by an independent investigator and remained concealed until completion of follow-up and database lock.

### 2.4. Data Collection and Analysis

The primary endpoint of the study was patient-reported pain-related symptom burden at 12 months, evaluated using the EQ-VAS. Secondary endpoints included: patient-reported pain-related symptom burden at 1, 2, 3, and 6 months, evaluated using the EQ-VAS; functional improvement, including quality of life and return to physical activity, evaluated using the KOOS; quadriceps muscle strength of both the treated and contralateral limbs, measured by a handheld dynamometer; the incidence of complications or adverse events; and the use of analgesics or nonsteroidal anti-inflammatory drugs (NSAIDs) throughout the study period.

### 2.5. Production of Platelet-Rich Plasma

PRP was produced as a clinical-grade blood component for non-transfusion use (Emocomponenti a uso non trasfusionale; EunT), in accordance with Italian Ministry of Health requirements for quality and safety standards for blood components (800–1200 × 10^9^/L) [40]. Quality control included verification of sterility, traceability, and platelet concentration release criteria, whereas growth factor analysis and cytokine profiling were not routinely performed.

#### 2.5.1. Cord Blood-Derived Platelet-Rich Plasma

Cord blood platelet concentrate (CBPC) units were obtained from allogeneic cord blood collected from placentas voluntarily donated to the cord blood bank by healthy mothers with term pregnancies after written informed consent. Cord blood units were collected into plastic bags containing 30 mL citrate phosphate dextrose adenine (CPDA-1) anticoagulant (JMS Singapore Pte. Ltd., Republic of Singapore) and processed within 48 h.

Cord blood underwent initial centrifugation at 210–240× *g* for 10 min (Cryofuge 6000i, r = 297 mm, Kendro Laboratory Products GmbH, Hanau, Germany). The resulting platelet-rich plasma (PRP) fraction was transferred to a secondary bag (CompoFlex^®^ 150 mL blood bag system, Fresenius Kabi AG, Bad Homburg, Germany) and centrifuged at 2000× *g* for 15 min. Excess platelet-poor plasma (PPP) was removed to obtain the final CBPC volume. The target volume was calculated using an automated Excel-based algorithm that accounted for PRP platelet concentration and the predefined platelet range required in the final product (800–1200 × 10^9^/L).

CBPC units were transferred to storage bags (PRP Secure^®^ PRPS-1 system, Biomed Device S.r.l., Modena, Italy) and cryopreserved without cryoprotectant at −80 °C in a mechanical freezer. Prior to clinical use, units were thawed at 37 °C in a water bath. PPP samples were cultured for aerobic and anaerobic bacteria and fungi using standard blood component culture procedures (BACT/ALERT^®^ pediatric aerobic and anaerobic culture bottles, bioMérieux, Marcy-l’Étoile, France).

Product safety was evaluated through serological testing of maternal and cord blood samples for infectious disease markers, including HIV-1/2, HCV, HBsAg, HBcAb, CMV, HTLV I–II, *Trypanosoma cruzi*, and *Treponema pallidum*, together with nucleic acid testing for HIV, HBV, and HCV. Cell counts and red blood cell immunophenotyping (ABO and Rh blood group) were also obtained from the initial material.

#### 2.5.2. Homologous Donor Platelet-Rich Plasma

HD-PRP was obtained from pooled buffy coats (BCs) derived from 450 mL whole blood units donated by repeat male donors with identical ABO blood groups. All donations tested negative for HIV, HBV, and HCV by both nucleic acid testing and serological screening.

Three BC units were pooled using a pooling kit with a leukocyte reduction filter (TF*FP06 10M1, Terumo Corporation, Tokyo, Japan) and diluted with plasma from one of the pooled units. Processing was performed in a closed system using a sterile connecting device (TSCD^®^-SC Sterile Tubing Welder, Terumo Corporation, Tokyo, Japan).

The BC pool was centrifuged at 275× *g* for 9 min, and the supernatant platelet concentrate was filtered into the storage bag. Platelet concentration in the resulting BC-derived platelet concentrate (BC-PC) was adjusted to 800–1200 × 10^9^/L by removing excess plasma after centrifugation at 2994× *g* for 18 min. Following a 60 min resting period, the platelet pellet was gently resuspended in an appropriate volume of plasma.

BC-PC units were stored overnight at 22 ± 2 °C on a flatbed agitator. The following day, samples were collected for platelet count and microbiological testing (BACT/ALERT^®^ pediatric aerobic and anaerobic culture bottles, bioMérieux, Marcy-l’Étoile, France). The BC-PC was then subdivided into 10 aliquots using a 10-bag system (PRP Secure^®^ PRPS-1 system, Biomed Device S.r.l., Modena, Italy) to obtain PRP, which was cryopreserved at −80 °C in a mechanical freezer for up to 5 years. Prior to clinical use, PRP aliquots were thawed at 37 °C in a water bath.

### 2.6. Clinical Evaluation

An initial physiotherapy assessment was performed before treatment initiation, followed by a series of standardized 30 min physiotherapy sessions throughout the 12-month follow-up period. At baseline and at each follow-up visit (1, 2, 3, 6, and 12 months), quadriceps muscle strength was assessed using a handheld dynamometer. Measurements were obtained for both the treated and contralateral limbs. Strength values are expressed in Newton per kilogram (N/kg), thereby normalizing muscle strength to body weight and allowing comparisons between individuals with different anthropometric characteristics.

Pain and functional status were evaluated prior to initial injection and at each follow-up visit using the EuroQol Visual Analog Scale (EQ-VAS) and the Knee Injury and Osteoarthritis Outcome Score (KOOS), respectively. The EQ-VAS is a 0–100 mm scale that records the patient’s self-perceived overall health status on the day of assessment, with 0 corresponding to the worst imaginable health state and 100 to the best imaginable health state; in patients with KOA, EQ-VAS scores are strongly influenced by pain severity and therefore provide a patient-reported measure of the overall impact of symptoms on health status [41,42]. The KOOS is a validated patient-reported outcome measure that evaluates knee health across five domains: pain, symptoms, activities of daily living, sport/recreational function, and knee-related quality of life [43]. Each domain is scored from 0 to 100, with higher scores indicating better function and fewer symptoms. Together, these measures provide a comprehensive assessment of both pain severity and functional status in patients with KOA.

At each follow-up visit, the use of analgesics or NSAIDs during the preceding month was recorded, together with any treatment-related adverse events or complications.

### 2.7. Radiological Evaluation

**Figure 1:**
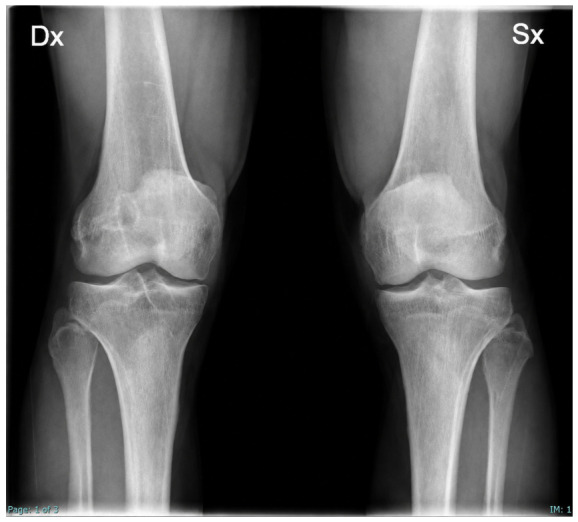
Radiograph of both knees demonstrating Kellgren–Lawrence grade 2 osteoarthritis, characterized by definite osteophyte formation, early joint space narrowing, and subchondral sclerosis of the tibial plateaus.

A preliminary radiological evaluation included an X-ray of the lower limbs in the orthostatic position, which was assessed according to the Kellgren–Lawrence scale for osteoarthritis (Figure 1).

### 2.8. Intervention

Patients were randomized to receive either CB-PRP or HD-PRP intra-articular knee injections. Injections were administered in a double-blind manner, with both patients and clinicians blinded to PRP type. Three injection cycles were performed at approximately 4-week intervals.

The first injection was performed under ultrasound guidance by experienced musculoskeletal radiologists, whereas the second and third injections were administered by orthopedic surgeons using an anatomy-guided technique based on established anatomical landmarks.

#### 2.8.1. Ultrasound-Guided Intra-Articular Injection Technique

The first PRP injection was performed by experienced musculoskeletal radiologists under ultrasound guidance using an in-plane technique to ensure optimal precision. A 12–15 MHz linear transducer (Philips, Singapore) was used. The procedure was performed with the patient in the supine position on the examination table, with the knee joint in extension or slight flexion (approximately 30°).

**Figure 2:**
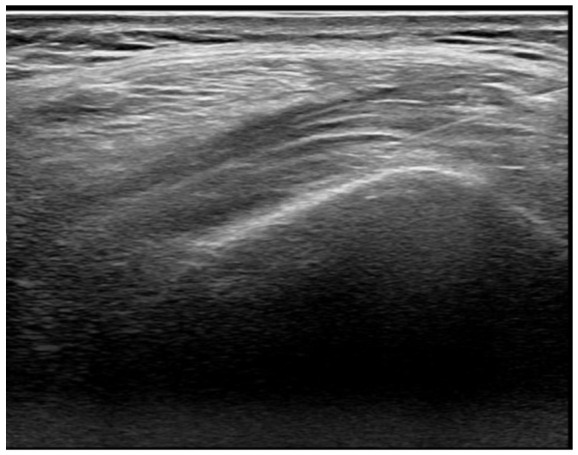
Ultrasound-guided intra-articular knee injection, with the needle visualized throughout its course using an out-of-plane technique, advancing into the subquadricipital recess.

A preliminary ultrasound examination was conducted to optimize focus, depth, and frequency settings. The sterile field was prepared using 2% chlorhexidine. Sterile conduction gel and a sterile probe cover were applied. The probe was positioned above the patella in an axial orientation. The skin was punctured at the lateral recess, and the needle was inserted at an angle of approximately 45°, advancing into the sub-quadricipital recess (Figure 2). Subsequently, 10 mL of PRP was injected using an 18-gauge needle.

After each injection, passive mobilization of the knee was performed, followed by the application of cryotherapy. Patients were then discharged with instructions to avoid prolonged activities and excessive loading for 1–2 days and were advised to continue applying ice on the injection site during the hours following the treatment.

#### 2.8.2. Anatomical Technique

The second and third PRP injections were performed by orthopedic surgeons using an anatomy-guided anterolateral approach based on palpable anatomical landmarks. The anterolateral portal was defined by the junction of the inferolateral border of the patella, the patellar tendon, and the lateral tibial plateau. For the anterolateral approach, the patient was positioned either seated or supine with the knee flexed at 90°, allowing improved exposure of the intra-articular surface, thus facilitating needle entry into the joint space.

Anatomic landmarks were first identified by palpation and marked with ink prior to the procedure, including the vastus lateralis, lateral femoral condyle, lateral tibial plateau, patellar tendon, and patella. A sterile needle was inserted lateral to the patellar tendon, approximately 1 cm above the tibial plateau, and advanced toward the intra-articular joint space at an angle of 15–45° relative to the vertical midline of the anterior knee surface.

### 2.9. Statistical Analysis

The primary endpoint of the study was defined as pain-related overall health perception at 12 months, measured using the EQ-VAS. To analyze the primary endpoint, a mixed model for repeated measures (MMRM) was employed, with the patient included as a random effect. The model included the change from baseline at 12 months as the dependent variable, treatment-by-visit interaction as fixed effects, and baseline score as a covariate.

Continuous secondary endpoints, including pain-related overall health perception at 1, 2, 3, and 6 months (EQ-VAS), quality of life and knee function (KOOS), and quadriceps muscle strength (dynamometer assessment), were analyzed using Student’s *t*-test for independent samples. Categorical variables, including incidence of complications and side effects and the use of analgesics or NSAIDs throughout the study period, were analyzed using Fisher’s exact test.

Statistical significance was defined as *p* < 0.05.

All analyses were conducted using R Statistical Software (version 4.3.1; R Core Team, Vienna, Austria).

## 3. Results

### 3.1. Population Characteristics

A total of 51 patients were enrolled, with 26 allocated to the CB-PRP group and 25 to the HD-PRP group. The mean age of the overall study population was 57.5 ± 5.3 years, with no significant difference between groups (*p* = 0.50). The proportion of female participants was significantly higher in the CB-PRP group (77%, 20/26) compared with the HD-PRP group (40%, 10/25, *p* = 0.017). No significant between-group differences were observed in body mass index (BMI), weight, height, or types of physical activity.

Table 1 illustrates the baseline population characteristics of the overall study cohort, stratified by treatment group.

### 3.2. Primary Endpoint: Pain-Related Symptom Burden Reduction at 12 Months

At 12 months, both groups demonstrated an overall reduction in pain, as measured by the EQ-VAS, with a consistent decrease from baseline (T0) observed throughout follow-up. In the CB-PRP group, the mean EQ-VAS score increased from 59 ± 19 at T0 to 83 ± 9 at the 12-month follow-up (T12). In the HD-PRP group, the mean EQ-VAS score started from 59 ± 18 at T0, reaching 77 ± 18 at T12. Although the CB-PRP group showed a slightly greater reduction in pain than the HD-PRP group, no significant difference was observed between the two groups (*p* = 0.46).

The number of evaluable patients varied slightly across time points due to intermittent missed assessments, as some participants did not attend specific follow-up visits but remained enrolled in the study. No permanent loss to follow-up occurred. At baseline (T0), data were available for all enrolled patients, while small amounts of missing data were observed at subsequent time points. At 12 months (T12), complete outcome data for EQ-VAS, KOOS, and quadriceps strength were available for 49 of 51 patients (96.1%).

Table 2 shows a mixed-effects model using change from baseline at T12 as the dependent variable, including treatment, visit, baseline score, and the visit-by-treatment interaction, which confirmed the main findings, with no significant interaction between visit and treatment (*p* = 0.55).

At T12, a total of 4 patients in the CB-PRP group and 5 patients in the HD-PRP group decided to drop out of the clinical trial and not to continue with follow-up due to personal problems.

### 3.3. Secondary Endpoints

At baseline (T0), the mean EQ-VAS score for the overall study population was 59 ± 18, with comparable values between the CB-PRP group (59 ± 19) and the HD-PRP group (59 ± 18), showing no statistically significant difference (*p* > 0.90). At the 1-month follow-up (T1), the mean EQ-VAS score increased to 67 ± 14 in the overall cohort, with slightly higher values observed in the CB-PRP group (68 ± 13) compared with the HD-PRP group (67 ± 16); however, the between-group difference was not statistically significant (*p* = 0.70). At the 2-month follow-up (T2), a further improvement was noted, with a mean EQ-VAS score of 75 ± 15; the CB-PRP group showed higher scores (78 ± 12) than the HD-PRP group (71 ± 17), although this difference, suggesting a trend, did not reach statistical significance (*p* = 0.14).

At the 3-month follow-up (T3), the overall mean EQ-VAS score increased to 79 ± 12, with the CB-PRP group reporting a mean score of 82 ± 9 compared with 76 ± 14 in the HD-PRP group; this difference approached statistical significance but remained below the predefined threshold, hence not sufficient to draw conclusions (*p* = 0.08).

At the 6-month follow-up (T6), the mean EQ-VAS score was 77 ± 16, with persistently higher values in the CB-PRP group (81 ± 11) compared with the HD-PRP group (74 ± 20), although no statistically significant difference was observed (*p* = 0.20). Finally, at the 12-month follow-up (T12), the mean EQ-VAS score further increased to 80 ± 14, with the CB-PRP group maintaining higher scores (83 ± 9) relative to the HD-PRP group (77 ± 18); again, the difference did not reach statistical significance (*p* = 0.20).

Table 3 presents the mean EQ-VAS scores at each time point for the CB-PRP and HD-PRP groups, along with the number of missing observations at each visit.

**Figure 3:**
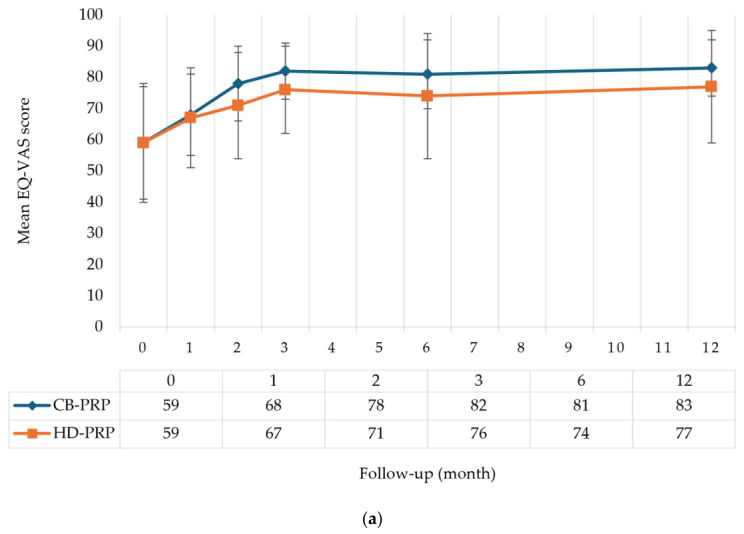

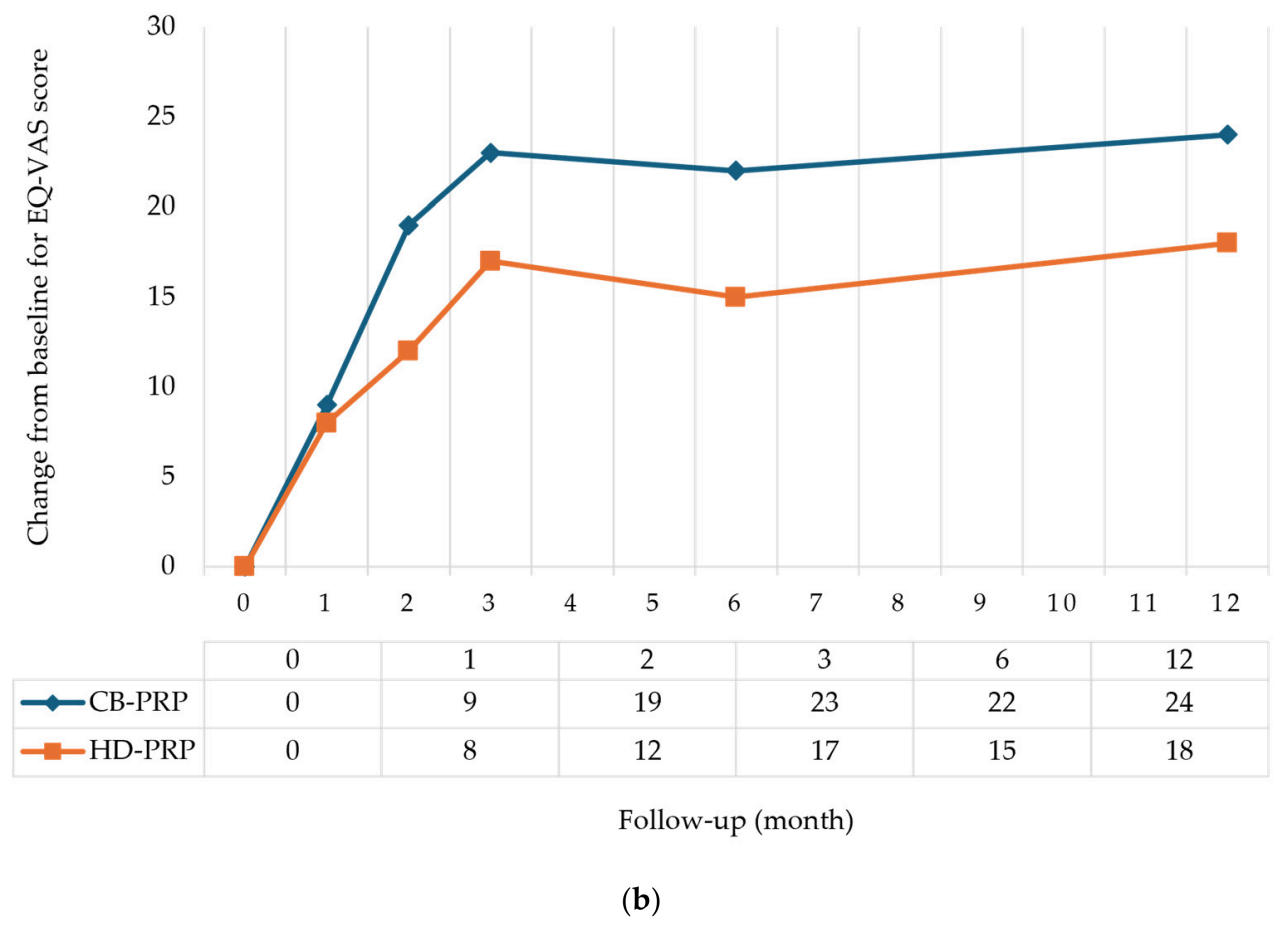
(**a**) Absolute EQ-VAS scores (mean ± standard deviation) for the CB-PRP and HD-PRP groups at months 0, 1, 2, 3, 6, and 12; (**b**) change from baseline in the EQ-VAS scores for the CB-PRP and HD-PRP groups at months 0, 1, 2, 3, 6, and 12.

Figure 3a illustrates the absolute EQ-VAS scores, while Figure 3b illustrates the change from baseline in EQ-VAS scores for the CB-PRP and HD-PRP groups at months 0, 1, 2, 3, 6, and 12.

Over time, KOOS values improved in both treatment groups, although the overall treatment effect was not statistically significant. At T0, the mean KOOS for the overall sample was 44 ± 27, with higher values in the CB-PRP group (50 ± 25) compared with the HD-PRP group (38 ± 28), a difference that was not statistically significant (*p* = 0.11). At T1, mean KOOS values increased to 55 ± 21 overall, with scores of 58 ± 21 in the CB-PRP group and 52 ± 22 in the HD-PRP group, again without a significant between-group difference (*p* = 0.30). This upward trend continued at T2, where the overall mean was 59 ± 23; the CB-PRP group scored 62 ± 22, and the HD-PRP group scored 55 ± 23, with no statistically significant difference observed (*p* = 0.20).

At T3, the overall mean KOOS was 63 ± 26, with higher scores in the CB-PRP group (69 ± 19) compared with the HD-PRP group (56 ± 31); this difference approached statistical significance (*p* = 0.094). At T6, the overall mean score was 64 ± 27, with the CB-PRP group scoring 72 ± 19 and the HD-PRP group maintaining higher scores at 56 ± 31; this between-group difference reached statistical significance (*p* = 0.036), indicating that the CB-PRP treatment had a more favorable outcome at this time point. At T12, the overall mean KOOS was 63 ± 24, with scores of 68 ± 23 in the CB-PRP group and 59 ± 24 in the HD-PRP group; however, the difference at this time point was no longer statistically significant (*p* = 0.20).

**Figure 4:**
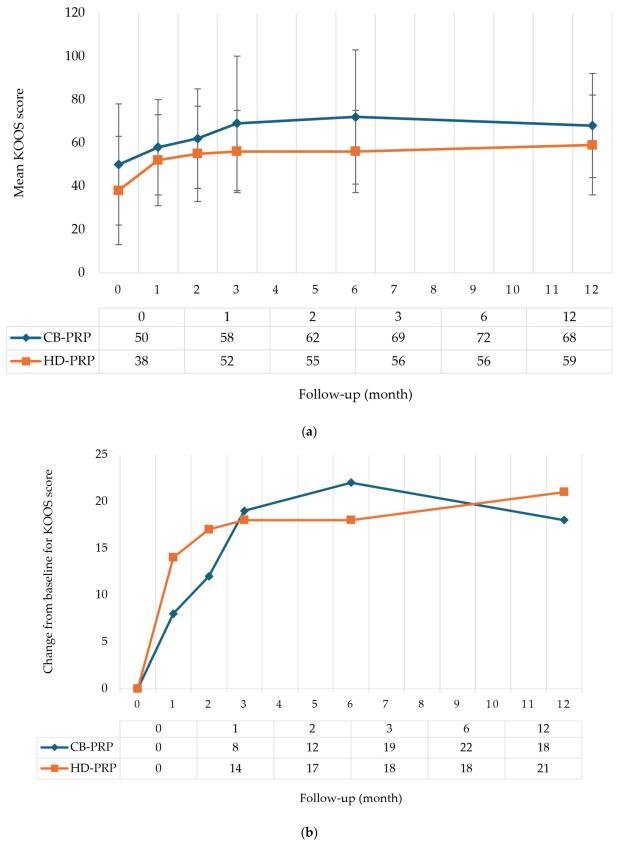
(**a**) Absolute KOOS (mean ± standard deviation) for the CB-PRP and HD-PRP groups at months 0, 1, 2, 3, 6, and 12; (**b**) change from baseline in the KOOS for the CB-PRP and HD-PRP groups at months 0, 1, 2, 3, 6, and 12.

Figure 4a illustrates the absolute KOOS, while Figure 4b illustrates the change from baseline in KOOS values for the CB-PRP and HD-PRP groups at months 0, 1, 2, 3, 6, and 12.

### 3.4. Quadriceps Muscle Strength

Quadriceps muscle strength was evaluated in both the treated and contralateral limbs throughout follow-up. Strength values, expressed in N/kg, showed progressive improvement over time in both treatment groups.

Table 4 reports complete numerical dynamometry results for both limbs.

In the treated limb, no statistically significant differences were observed between the CB-PRP and HD-PRP groups at baseline or at any follow-up assessment (*p* > 0.05). At the 12-month follow-up, quadriceps strength remained comparable between groups (*p* = 0.22). In the contralateral limb, a statistically significant between-group difference was observed only at the 2-month follow-up (*p* = 0.038). However, this isolated finding was not maintained at subsequent follow-up visits and was considered of limited clinical relevance.

Quadriceps muscle strength was then analyzed separately for the treated and contralateral limbs using MMRM. For the treated limb, quadriceps strength improved significantly over time (*p* = 0.0004). However, no significant treatment effect (*p* = 0.85) or treatment-by-visit interaction (*p* = 0.22) was observed, indicating that changes in quadriceps strength over time were comparable between the CB-PRP and HD-PRP groups. For the contralateral limb, neither the effect of time (*p* = 0.09) nor the treatment-by-visit interaction (*p* = 0.41) was statistically significant. Although a significant main effect of treatment group was observed (*p* = 0.04), no significant between-group differences were detected in the longitudinal changes in quadriceps muscle strength.

Table 5 presents complete MMRM results for both limbs.

### 3.5. Adverse Reactions to Infiltration

At baseline (T0), adverse reactions were reported in 9 patients (34.6%) in the CB-PRP group and 7 patients (28.0%) in the HD-PRP group, corresponding to an overall prevalence of 16 patients (31.4%). In both groups, only mild local reactions were observed at the initial post-injection assessment, including joint stiffness, slight swelling, or localized edema. All reactions resolved spontaneously and were not reported at subsequent follow-up visits. No significant difference between groups was observed (*p* = 0.80).

At the 1-month follow-up (T1), adverse reactions were reported in 5 patients (19.2%) in the CB-PRP group and 4 patients (16.0%) in the HD-PRP group (overall prevalence: 17.6%), with no significant difference between groups (*p* > 0.90).

At 2 months (T2), adverse reactions were reported in 3 patients (11.5%) in the CB-PRP group and 2 patients (8.0%) in the HD-PRP group (overall prevalence: 9.8%). Again, no statistically significant difference was observed between groups (*p* > 0.90).

### 3.6. Use of Analgesics or Nonsteroidal Anti-Inflammatory Drugs

At baseline (T0), approximately 49% of enrolled patients reported the use of analgesics or NSAIDs, with similar proportions in the CB-PRP (50%) and HD-PRP (48%) groups (*p* > 0.90). During follow-up, analgesic use progressively declined in both treatment groups, reflecting the overall clinical improvement observed after PRP treatment.

The greatest reduction was observed at the 3-month follow-up (T3), when 94% of CB-PRP patients and 85% of HD-PRP patients reported complete discontinuation of analgesic use. Thereafter, a slight increase in medication use was observed in both groups, although analgesic consumption remained substantially lower than at baseline throughout the remainder of the follow-up period.

By the 12-month follow-up (T12), 78% of the overall study population reported no need for analgesic medication, corresponding to 76% of patients in the CB-PRP group and 69% in the HD-PRP group. No statistically significant differences in analgesic use were observed between the two treatment groups throughout the study period (*p* > 0.05).

Analysis of consumption frequency showed that at baseline (T0), 24% of CB-PRP patients and 31% of HD-PRP patients reported no analgesic use, while approximately half of the remaining participants in both groups reported taking analgesics two to five times during the previous month. Overall analgesic or NSAID use during the study period was comparable between groups, reported by 17 patients (65.4%) in the CB-PRP group and 16 patients (64.0%) in the HD-PRP group, corresponding to an overall prevalence of 64.7%, with no significant between-group differences (*p* > 0.90).

Regarding medication type, non-selective COX-1 inhibitors were the most frequently reported agents at baseline: 70.6% in the CB-PRP group and 62.5% in the HD-PRP group. Use of COX-2 inhibitors and paracetamol remained minimal and stable across visits. The predominance of COX-1 inhibitor use persisted throughout follow-up, indicating that traditional NSAIDs were the preferred symptomatic treatment when pharmacological therapy was required.

## 4. Discussion

The present single-center, randomized, double-blind pilot clinical trial investigated the comparative efficacy and safety of two standardized PRP formulations—umbilical CB-PRP and HD-PRP—in patients with early-stage KOA. Overall, both treatments resulted in comparable improvements in pain, knee function, and quadriceps muscle strength over the 12-month follow-up period. Although CB-PRP demonstrated numerically higher EQ-VAS and KOOS values at several follow-up visits, these differences were modest and generally failed to reach statistical significance, with the exception of a transient advantage in KOOS at 6 months. Overall, our findings indicate that the enhanced biological profile previously described for CB-PRP did not translate into clinically meaningful superiority over HD-PRP during the first year after initiation of treatment.

These findings are consistent with the growing evidence supporting the clinical efficacy of PRP in patients with early-stage KOA. Recent studies have demonstrated that PRP provides greater and more durable improvements in pain and function than HA and placebo, particularly in patients with mild-to-moderate disease. Yi et al. (2025) evaluated 29 systematic reviews and meta-analyses investigating PRP for KOA and concluded that, although the overall evidence consistently supports favorable clinical outcomes, the methodological quality of the available reviews remains predominantly low, with substantial overlap among primary studies and considerable heterogeneity in PRP preparation protocols [44]. Consequently, while the beneficial effects of PRP appear well established, uncertainty persists regarding the relative efficacy of different PRP formulations. The present study contributes to addressing this knowledge gap by comparing two standardized allogeneic platelet products manufactured under identical blood bank conditions.

Pain relief represented one of the most relevant clinical outcomes of the present study. Both CB-PRP and HD-PRP produced progressive reductions in pain during follow-up, with maximal improvement observed between three and six months and maintenance of clinical benefit at one year. This temporal pattern closely resembles that reported in previous investigations evaluating PRP for KOA. In particular, Coviello et al. (2025) compared CB-PRP with conventional autologous PRP in patients with mild-to-moderate KOA and similarly observed a transient advantage of CB-PRP during the first three to six months after treatment [11]. However, this difference progressively diminished, and no significant differences in pain scores were observed after 12 months. Likewise, Mazzotta et al. (2022) reported that CB-PRP produced early clinical improvement in patients with hip osteoarthritis without demonstrating sustained superiority over autologous PRP during longer follow-up [45]. Taken together with the present findings, these studies consistently suggest that although CB-PRP may induce greater early symptomatic improvement, its long-term analgesic efficacy appears comparable to that of other PRP formulations.

Functional recovery followed a pattern similar to pain improvement. Both treatment groups demonstrated progressive increases in KOOS throughout follow-up, with the greatest improvements occurring during the first six months after injection. Although CB-PRP showed significantly better KOOS values at the six-month assessment, this advantage was no longer evident at the final follow-up visit. These observations closely mirror those of Coviello et al. (2025), who reported greater improvements in WOMAC (Western Ontario and McMaster Universities) and KOOS during the early follow-up period after CB-PRP administration but found no clinically meaningful differences compared with autologous PRP after one year [11]. Similarly, previous studies comparing different biologic intra-articular therapies have generally failed to demonstrate durable superiority of one platelet preparation over another, suggesting that once an effective biological stimulus has been delivered, long-term clinical outcomes may depend more on patient-related factors and disease biology than on the specific platelet source.

The absence of sustained clinical superiority of CB-PRP is particularly interesting considering its reported biological characteristics. Experimental studies have consistently demonstrated that CB-PRP contains higher concentrations of several regenerative growth factors and anti-inflammatory cytokines than adult peripheral blood-derived platelet preparations. Mani et al. (2024) showed that CB-PRP contains significantly greater concentrations of growth factors, enhances cell proliferation and migration, promotes macrophage polarization toward an anti-inflammatory phenotype, and reduces IFN-γ production compared with adult-derived PRP [39]. These findings provide a strong biological rationale for the use of CB-PRP as a regenerative therapy and support the hypothesis that it may possess greater anti-inflammatory potential than conventional platelet preparations. Likewise, previous laboratory investigations have reported higher concentrations of IL-10 and other regenerative mediators in CB-PRP, together with enhanced cellular proliferation and extracellular matrix synthesis.

Despite these promising experimental findings, our clinical results suggest that enhanced biological activity does not necessarily translate into superior long-term clinical outcomes. Several mechanisms may explain this apparent discrepancy. First, the therapeutic effects of PRP depend not only on the concentration of individual growth factors but also on the complex interaction among platelets, synovial tissue, cartilage, subchondral bone, immune cells, and the inflammatory microenvironment of the osteoarthritic joint. Second, it is plausible that both HD-PRP and CB-PRP provide concentrations of bioactive mediators above the threshold required to induce a therapeutic response. Once this threshold has been reached, further increases in growth factor concentration may produce diminishing clinical returns, resulting in a biological “ceiling effect” [46,47]. Finally, osteoarthritis is a multifactorial disease influenced by mechanical loading, metabolic factors, inflammatory pathways, and patient-specific characteristics, all of which may attenuate the impact of differences in platelet composition.

The present findings should also be interpreted within the context of current clinical evidence regarding allogeneic PRP. Most published clinical studies have evaluated autologous PRP, whereas relatively few have investigated donor-derived platelet products. Consequently, evidence comparing different allogeneic preparations remains limited. Our study therefore expands the available literature by demonstrating that two standardized allogeneic PRP formulations with different biological origins produce comparable improvements in pain, function, and quality of life. Together with the findings reported by Coviello et al. (2025), our results suggest that although CB-PRP possesses theoretical biological advantages, current clinical evidence does not support a clear long-term superiority over either autologous or homologous donor-derived PRP [11].

Autologous PRP remains the most commonly used PRP formulation in routine orthopedic practice because it can be prepared directly from the patient’s own blood, is immediately available, avoids donor exposure, and is widely accepted in clinical practice. Consequently, direct comparison with autologous PRP would undoubtedly have enhanced the immediate translational relevance of the present study. Nevertheless, the primary objective of the present study was fundamentally different. Rather than reassessing the efficacy of PRP itself, which has already been extensively investigated, we sought to compare two standardized allogeneic platelet products characterized by distinct biological origins and molecular profiles but manufactured under identical blood bank conditions. This design minimized the substantial variability commonly associated with autologous PRP, which may be influenced by age, sex, baseline platelet count, medications, systemic inflammation, comorbidities, and preparation techniques [48]. By reducing these confounding factors, we were able to specifically investigate whether the source of donor platelets influenced clinical outcomes. Future adequately powered randomized trials directly comparing autologous PRP, HD-PRP, and CB-PRP will be essential to determine their relative efficacy, safety, cost-effectiveness, and optimal clinical indications.

Safety outcomes were reassuring. Both platelet preparations were well tolerated, with only mild transient local reactions reported shortly after injection. Although allogeneic products may raise concerns regarding the potential transmission of infectious diseases, both adult blood donors and umbilical cord blood units undergo rigorous donor selection and mandatory screening for transfusion-transmissible infections in accordance with national transfusion regulations [49]. Cord blood units that do not meet eligibility and safety requirements are excluded from clinical use. In addition, all PRP preparations were produced, processed, and stored according to validated blood bank procedures, ensuring high standards of quality and safety [50]. Consistent with these safeguards, no treatment-related infections, severe adverse events, or unexpected complications occurred during follow-up. Analgesic and NSAID consumption progressively decreased in both treatment groups, further supporting the sustained symptomatic improvement observed throughout the study.

From a clinical perspective, our findings have several practical implications. Unlike autologous PRP, standardized allogeneic platelet products can be manufactured under controlled blood bank conditions, ensuring rigorous quality control, reproducible platelet concentration, sterility, traceability, and standardized storage protocols. For instance, both CB-PRP and HD-PRP were stored at −80 °C under validated conditions; previous studies on allogeneic platelet derivatives have shown that cryopreservation preserves the biological activity and growth factor content of these products [51,52]. This approach also addresses the issue that platelet concentration has been reported as a potential determinant of PRP efficacy [53]. Such standardization substantially reduces the biological variability that frequently complicates the interpretation of studies involving autologous PRP.

Furthermore, cord blood units that are unsuitable for hematopoietic stem cell transplantation because of insufficient volume or cellularity may still represent valuable sources for PRP production, thereby improving the utilization of donated biological material [54]. Batch manufacturing also enables the production of multiple therapeutic doses from a single donation, potentially improving production efficiency and facilitating multicenter clinical implementation. Nevertheless, these potential advantages must be balanced against the additional logistical requirements and regulatory oversight associated with donor-derived biological products. Since current clinical evidence has not demonstrated clear long-term superiority of CB-PRP, treatment selection should presently consider not only biological characteristics but also manufacturing feasibility, availability, regulatory considerations, and cost-effectiveness.

The present study has several strengths. Both PRP formulations were prepared using validated blood bank procedures under standardized manufacturing conditions, minimizing product variability and ensuring reproducibility. The randomized, double-blind design reduced selection and observer bias, while the prospective 12-month follow-up allowed comprehensive evaluation of pain, function, muscle strength, quality of life, medication use, and safety.

Nevertheless, several limitations should be acknowledged. First, the relatively small sample size may have limited statistical power to detect subtle but potentially relevant differences between treatments. Second, the unequal sex distribution between groups—a higher proportion of female participants in the CB-PRP group (*p* = 0.017)—may have introduced residual confounding despite randomization, given known sex-related differences in pain perception and osteoarthritis symptom reporting [55,56]. Third, the absence of autologous PRP and placebo or HA comparator groups limits conclusions regarding the relative performance of these formulations in routine clinical practice and precludes assessment of the absolute efficacy of either treatment. Finally, structural disease progression was not evaluated using imaging modalities such as magnetic resonance imaging, and therefore no conclusions can be drawn regarding cartilage regeneration or disease modification.

Future RCTs should incorporate standardized PRP characterization, harmonized reporting criteria, adequately powered multicenter designs, imaging-based structural outcomes, and direct comparisons among autologous, HD-PRP, and CB-PRP to generate higher-quality evidence and improve the clinical applicability of PRP therapy for KOA.

## 5. Conclusions

Both CB-PRP and HD-PRP demonstrated sustained improvements in pain and function in patients with early-stage KOA over a 12-month follow-up period, with no statistically significant differences between treatments. Quadriceps muscle strength also improved over time in both groups without significant between-group differences in either the treated or contralateral limb.

Both preparations were safe, well-tolerated, and produced via standardized, reproducible protocols, ensuring consistent, reproducible preparation. Although CB-PRP showed non-significant trends toward better outcomes, it did not demonstrate superiority over HD-PRP, indicating comparable clinical outcomes.

These findings do not support a differential clinical advantage of either preparation and should be interpreted in the context of the pilot design and limited sample size. Further larger, adequately powered studies with extended follow-up are required to confirm these results and better clarify the comparative effectiveness of allogeneic PRP formulations in KOA.

## Figures and Tables

**Table 1:** Population characteristics by treatment group.

Population Characteristics	Cord (*n* = 26)	Homologous (*n* = 25)	*p*-Value
Age (y)	57.0 ± 5.6	58.0 ± 5.0	0.50
Age category (y)			0.60
<56	8/26 (31%)	9/25 (36%)	0.60
56–60	13/26 (50%)	9/25 (36%)	0.60
>60	5/26 (19%)	7/25 (28%)	0.60
Gender			0.017
Female sex (*n*, %)	20/26 (77%)	10/25 (40%)	0.017
Weight (kg)	71 ± 15	77 ± 13	0.20
Height (cm)	170 ± 9	173 ± 10	0.30
Body Mass Index (kg/m^2^)	24.27 ± 3.51	25.56 ± 2.82	0.20
Physical activity			0.30
Sedentary	4/26 (15.38%)	6/25 (24.00%)	0.30
Moderate	15/26 (57.69%)	12/25 (48.00%)	0.30
Intense	7/26 (26.92%)	6/25 (24.00%)	0.30

**Table 2:** Mixed models for repeated measures (MMRM) for change from baseline, including treatment, visit, baseline score, and treatment-by-visit interaction.

Fixed Effect	Estimate	*p*-Value
Intercept	70.36 (5.84)	<0.001
Visit	0.72 (0.23)	0.0016
Treatment	4.10 (3.58)	0.26
Baseline	−1.01 (0.09)	<0.001
Visit × Treatment	0.19 (0.32)	0.55

**Table 3:** Mean EQ-VAS scores at each time point presented for the CB-PRP and HD-PRP groups, with the number of missing observations reported for each visit.

Mean EQ-VAS Score			
Time Point	Cord (*n* = 26)	Homologous (*n* = 25)	*p*-Value
T0	59 ± 19	59 ± 18	>0.90
(Missing)	0	0	
T1	68 ± 13	67 ± 16	0.70
(Missing)	0	1	
T2	78 ± 12	71 ± 17	0.14
(Missing)	1	0	
T3	82 ± 9	76 ± 14	0.08
(Missing)	1	1	
T6	81 ± 11	74 ± 20	0.20
(Missing)	1	2	
T12	83 ± 9	77 ± 18	0.20
(Missing)	1	1	

**Table 4:** Quadriceps muscle strength (N/kg) of the treated and contralateral limbs in the CB-PRP and HD-PRP groups at baseline and follow-up time points.

	Treated Limb			Contralateral Limb		
Time Point	CB-PRP	HD-PRP	*p*-Value	CB-PRP	HD-PRP	*p*-Value
T0	3.18 ± 0.71	3.22 ± 0.69	0.82	3.54 ± 0.66	3.49 ± 0.68	0.79
T1	3.32 ± 0.68	3.30 ± 0.71	0.91	3.61 ± 0.63	3.55 ± 0.66	0.74
T2	3.47 ± 0.66	3.39 ± 0.69	0.67	3.84 ± 0.58	3.48 ± 0.61	0.038
T3	3.61 ± 0.64	3.55 ± 0.67	0.72	3.79 ± 0.60	3.67 ± 0.64	0.46
T6	3.73 ± 0.63	3.69 ± 0.66	0.81	3.83 ± 0.59	3.75 ± 0.63	0.63
T12	3.76 ± 0.65	3.73 ± 0.68	0.22	3.82 ± 0.61	3.78 ± 0.65	0.77

**Table 5:** Mixed models for repeated measures (MMRM) results for quadriceps muscle strength in the treated and contralateral limbs.

Fixed Effect	Treated Limb Estimate (SE)	*p*-Value	Contralateral Limb Estimate (SE)	*p*-Value
Intercept	76.82 (20.65)	0.0005	61.76 (20.05)	0.0033
Visit	3.21 (0.90)	0.0004	1.58 (0.92)	0.09
Treatment	−2.74 (14.57)	0.85	−27.71 (13.15)	0.04
Baseline	−0.187 (0.062)	0.0040	−0.111 (0.061)	0.07
Visit × Treatment	−1.52 (1.24)	0.22	1.05 (1.28)	0.41

## Data Availability

The raw data supporting the conclusions of this article will be made available by the authors on request.

## Abbreviations

The following abbreviations are used in this manuscript:

BC	Buffy Coat
BC-PC	Buffy Coat-Derived Platelet Concentrate
BMI	Body Mass Index
BMP	Bone Morphogenetic Proteins
CB-PC	Cord Blood Platelet Concentrate
CB-PRP	Cord Blood-Derived Platelet-Rich Plasma
CMV	Cytomegalovirus
CPDA	Citrate Phosphate Dextrose Adenine
EQ-VAS	EuroQol Visual Analogue Scale
HA	Hyaluronic Acid
HBcAb	Hepatitis B Core Antibody
HBsAg	Hepatitis B Surface Antigen
HCV	Hepatitis C Virus
HD-PRP	Homologous Donor Platelet-Rich Plasma
HIV	Human Immunodeficiency Virus
HTLV	Human T-Lymphotropic Virus
IFN-γ	Interferon-Gamma
IGF	Insulin-like Growth Factor
IL	Interleukin
KOA	Knee Osteoarthritis
KOOS	Knee Injury and Osteoarthritis Outcome Score
MMRM	Mixed Model for Repeated Measures
MRI	Magnetic Resonance Imaging
NSAID	Nonsteroidal Anti-Inflammatory Drug
OA	Osteoarthritis
PDGF	Platelet-Derived Growth Factor
PPP	Platelet-Poor Plasma
PRP	Platelet-Rich Plasma
RCT	Randomized Controlled Trial
TGF	Transforming Growth Factor
VEGF	Vascular Endothelial Growth Factor
